# Replacement and Immunomodulatory Activities of 20% Subcutaneous Immunoglobulin Treatment: A Single-Center Retrospective Study in Autoimmune Myositis and CVID Patients

**DOI:** 10.3389/fimmu.2021.805705

**Published:** 2022-01-17

**Authors:** Maria Giovanna Danieli, Jacopo Umberto Verga, Cristina Mezzanotte, Irene Terrenato, Silvia Svegliati, Maria Beatrice Bilo, Gianluca Moroncini

**Affiliations:** ^1^ Department of Clinical and Molecular Sciences, Marche Polytechnic University, Ancona, Italy; ^2^ Clinica Medica, Department of Internal Medicine, Ospedali Riuniti, Ancona, Italy; ^3^ Department of Life and Environmental Sciences, Polytechnic University of Marche, Ancona, Italy; ^4^ The Science Foundation Ireland (SFI) Centre for Research Training in Genomics Data Science, National University of Ireland, Galway, Ireland; ^5^ Internal Medicine Residency Program, Marche Polytechnic University, Ancona, Italy; ^6^ Biostatistics and Bioinformatic Unit, Scientific Direction, IRCCS Regina Elena Cancer Institute, Rome, Italy; ^7^ Allergy Unit, Department of Internal Medicine, Ospedali Riuniti, Ancona, Italy

**Keywords:** common variable immunodeficiency, immunomodulation, intravenous immunoglobulin, polymyositis, dermatomyositis

## Abstract

**Background:**

Immunoglobulin (Ig) replacement therapy represents a life-saving treatment in primary antibody deficiencies. The introduction of subcutaneous Ig (SCIg) administration brings a major improvement in quality of life for patients, compared to the traditional intravenous administration. In recent years, an additional role has been proposed for Ig therapy for various inflammatory and immune-mediated diseases. Consequently, the use of SCIg has expanded from immunodeficiencies to immune-mediated diseases, such as polymyositis (PM) and dermatomyositis (DM). Given the rarity of these conditions, it is still difficult to evaluate the real impact of SCIg treatment on PM and DM, and additional data are constantly required on this topic, particularly for long-term treatments in real-life settings.

**Aim:**

This study aimed to increase the knowledge about the anti-inflammatory and immunomodulatory effects of SCIg treatment for myositis. To this aim, a long-term evaluation of the effectiveness of 20% human SCIg treatment (20% SCIg, Hizentra^®^, CSL Behring) was carried out in patients with PM/DM in care at our Center. In addition, an evaluation of the 20% SCIg therapy in CVID patients was provided. This analysis, beside adding knowledge about the use of SCIg therapy in this real-life setting, was intended as a term of comparison, regarding the safety profile.

**Results:**

Results support the beneficial effect and tolerability of long-term 20% SCIg therapy in PM/DM patients, reporting a significant improvement in creatine kinase levels, muscle strength, skin conditions, dysphagia, disease activity (MITAX score) and disability (HAQ-DI score). None of the patients reported systemic reactions. The duration of the reported local reactions was a few hours in 80% of the patients, and all resolved spontaneously. CVID patients reported an improvement in all the considered effectiveness parameters at the end of 20% SCIg therapy. The frequency of the adverse events reported by PM/DM patients was not different from what reported in CVID patients, where the use of SCIg therapy is more consolidated

**Conclusions:**

This study suggests that 20% SCIg treatment represents a viable and safe treatment for PM/DM patients and a valid therapeutic alternative to IVIg, with important advantages for patients’ quality of life.

## 1 Introduction

Immunoglobulin (Ig) administered through intravenous injection (IVIg) represented a lifesaving therapy in primary antibody deficiencies (1–5). More recently, subcutaneous Ig (SCIg) administration has become available, bringing a significant improvement in terms of quality of life for patients (6–9). Indeed, SCIg does not require venous access and is associated with more stable serum IgG levels, is able to potentially reduce the “wear-off effect” and presents a lower incidence of systemic adverse events (AEs) (6–13).

In recent years, an additional role for Ig therapy in the treatment of various inflammatory and immune-mediated diseases has been proposed (14, 15). Consequently, although the related mechanisms of action are complex and only partially understood, the use of SCIg treatment has expanded from immunodeficiencies to autoimmune diseases, as polymyositis (PM) and dermatomyositis (DM) (16–19).

PM and DM are idiopathic immune-mediated myopathies characterized by inflammation and weakness of proximal muscles with extra muscular manifestations (20–22). Besides the involvement of skin, high serum creatinine kinase (CK) levels, serum autoantibodies, inflammatory infiltrates in skeletal muscle, and some peculiar features in electromyography and MRI characterize DM and PM patients (21–24).

A correct diagnosis and an early initiation of therapy are essential in these conditions (23). Dalakas firstly reported the efficacy of IVIg in patients with DM (25). Afterward, additional data supported IVIg to control muscular disease activity and improve muscular strength in patients with PM and DM, and an increased survival in patients treated with SCIg compared with older series published in the 1990s also documented (18). Nevertheless, given the rarity of these conditions, it is still difficult to evaluate the real impact of SCIg treatment on PM and DM and additional data are constantly required on this topic, particularly for long-term treatments in real-life settings.

Ours is a referral center for patients affected by autoimmune disorders and immunodeficiency diseases. From November 2011, a 20% human IgG product for subcutaneous administration (Hizentra^®^, CSL Behring GmbH, Marburg, Germany; hereafter termed 20% SCIg) has been available in Italy and was introduced as reference therapy for patients in care at our center. Compared to other SCIg products, its characteristics of high-level purity (>98% IgG), higher IgG content (20%) and reduced viscosity (14.7 ± 1.2 mPas) enable a low infusion volumes and high infusion rates (26), thus representing an improved SCIg option.

To increase the knowledge about the anti-inflammatory and immunomodulatory effects of SCIg treatment for myositis, we report our experience in a real-life, long-term evaluation of benefit and safety of 20% SCIg treatment in patients with PM/DM. In addition, we provide an evaluation of 20% SCIg therapy in CVID patients. This analysis, beside adding knowledge about the use of SCIg therapy in this real-life setting, was intended as a term of comparison, regarding the safety profile.

## 2 Patients and Methods

### 2.1 Study Design and Setting

This was a single-center retrospective study, carried out at the Clinica Medica, Ospedali Riuniti Ancona and Marche Polytechnic University (central Italy), which is a member of MyoNet (a global, multicenter, interdisciplinary research project on inflammatory myopathies), a regional referral center for IPINet (Italian Primary Immunodeficiencies Network) and Documenting Centre for European Society for Immunodeficiencies (ESID) (27). All patients’ data were analyzed from the dedicated database. Patients who underwent at least one cycle of 20% SCIg treatment and followed-up for more than 1 year were included in the study (cut-off date: June 2021). Patients who initiated a SCIg therapy before 2011 were started on 16% SCIg (Vivaglobin^®^, CSL Behring GmbH, Marburg, Germany) and then switched to 20% SCIg therapy (28).

The study was notified to the Ethics Committee of Marche Region and was performed in accordance with the International Conference on Harmonization Good Clinical Practice guidelines and the Declaration of Helsinki. All patients previously gave informed consent to demographic, clinical and laboratory data collection and publication (Protocol number: 2012 212024 OR of 02/02/2012; n. 138/DG 20/03/2012 for myositis patients, protocol number: 2016 0561 OR of 27/10/2016; n. 871 DG 7/12/2016 for CVID patients, AOU Ospedali Riuniti, Ancona, Italy).

### 2.2 PM and DM Patients

The diagnosis of PM/DM was made according to Bohan and Peter’s criteria and confirmed in agreement with the new diagnostic criteria of the European League Against Rheumatism/American College of Rheumatology (EULAR/ACR) (29). The 20% SCIg treatment was administered at the weekly dose of 0.2 g/kg, according to the procedures previously described (30).

#### 2.2.1 Study Assessments

All study measures were assessed before (pre-treatment values) and after (post-treatment values) 20% SCIg therapy.

The routine procedure comprises a general physical examination with emphasis on the muscle and the skin. The muscle evaluation was based on the manual muscle test 8 (MMT8), which assesses changes in skeletal muscle strength in six proximal and two distal muscular districts, with a score range of 0–10 for each tested muscle (31). As a biochemical index of muscle damage, we collected data related to CK (normal values <170 U/L). As a working definition, complete skeletal muscle remission was defined in the presence of MMT8 values ≥78 with normal serum CK levels. Partial remission was present when only one of the above criteria was satisfied.

The immunological parameters included antinuclear antibodies and anti-extractable nuclear antigen antibodies by immunoblotting analysis to detect the different patterns. Testing for serum myositis-specific autoantibodies and myositis-associated autoantibodies has been performed by immunoblotting (Alphadia, Belgium).

Lung function was assessed through the diffusing capacity of the lung to carbon monoxide (DLCO) and the forced vital capacity (FVC) evaluations.

All patients underwent a complete cardiologic evaluation, including an echocardiographic examination.

The presence and severity of dysphagia were quantified using the Dysphagia Outcome and Severity Scale (DOSS), a 7-point scale from 1 (severe dysphagia) to 7 (normal in all situations).

In all patients, the presence of underlying malignancies was investigated. We also collected data related to the treatment with glucocorticoids and immunosuppressive agents and side effects.

##### 2.2.1.1 Assessment of Disease Activity

The disease activity, defined as potentially reversible and related only to the myositis disease process, was evaluated with the Myositis Intention to Treat Activities Index (MITAX). MITAX explores the disease activity in seven organ systems (constitutional, cutaneous, skeletal, gastrointestinal, pulmonary, cardiac and muscle). According to the degree of inflammation, each clinical manifestation is calculated from 0 to 4 (not present – new feature). The summed score is then divided by the maximum possible score. Higher scores reflect a more severe activity (32).

##### 2.2.1.2 Assessment of Damage

The myositis damage index (MDI) score was used to evaluate persistent changes in 9 organ systems (muscular, skeletal, cutaneous, gastrointestinal, pulmonary, cardiovascular, peripheral vascular, endocrine, and ocular) plus infections and malignancies. Each scale comprises 2–8 items scored as present (if persisting for at least 6 months) or absent. The scores were summed to provide a total MDI damage score (potential range: 0–38 in adults). The total MDI of each patient was normalized for the number of items considered for the single patient to obtain MDI values comparable to each other (32, 33).

##### 2.2.1.3 Assessment of Disability

The Health Assessment Questionnaire related to physical disability (HAQ-DI) comprises 20 questions investigating eight activities: dressing and grooming, arising, eating, walking, hygiene, reaching, gripping. The HAQ-DI is graded from 0 (no difficulty) to 3 (unable to do). Responses in at least six of the eight categories are necessary. The highest sub-category score determines the value for each category; the HAQ-DI is then computed by dividing the summed component scores by the number of components answered. Disability was classified as moderate to severe with a HAQ-DI score ≥1.0.

### 2.3 CVID Patients

CVID was diagnosed according to the revised ESID criteria (34), and/or according to the International Consensus Document criteria, for cases preceding ESID 2019 revision (35, 36). The clinical phenotypes of patients were classified according to the work of Chapel and collaborators (37). The 20% SCIg treatment was administered every 7–10 days at a 0.2 g/kg/weekly dose.

#### 2.3.1 Study Assessments

The following parameters were evaluated before and after 20% SCIg treatment: serum IgG trough levels, the number of infection episodes (serious and non-serious), the number of days out of work, the number of days hospitalized due to infections, the duration of antibiotic use for infection prophylaxis and treatment. Safety data were also collected and compared between PM/DM and CVID patients.

### 2.4 Patients’ Satisfaction

All patients were asked to respond to a quick satisfaction survey composed of six questions about their personal experience with 20% SCIg treatment.

### 2.5 Statistical Analyses

All variables of interest were summarized by descriptive statistics. Categorical data were presented as frequencies and percentage values while continuous variables as median values and their relative range. The Wilcoxon and McNemar non-parametric tests were applied to test efficacy indicators before and after SCIg therapy administration, when appropriate. The Mann-Whitney non-parametric test was used to compare independent groups. A p<0.05 was considered statistically significant. All Analyses were carried out with SPSS (SPSS version 21.0, IBM, Armonk, NY, USA).

## 3 Results

### 3.1 PM and DM Patients

Overall, data from 30 PM/DM patients were analyzed. **Table 1** summarizes the baseline characteristics of these patients.

**Table 1:** Baseline characteristics of PM and DM patients (n = 30).

	n	%
Age at diagnosis (years), median (min–max)	58 (18–77)	
Gender: Females	24	80
Type of myositis		
PM	16	54
DM	14	46
Autoantibodies positivity:		
Antinuclear antibodies	13	43
Anti-SRP	3	10
Anti-Jo1	3	10
Anti-Mi-2	3	10
Anti-MDA-5	1	3
Anti-myositis-associated autoantibodies (SSA, SSB, RNP)	6	20
Previous IVIg treatment	19	63
Other therapies		
Oral prednisone/methylprednisolone	29	100
Hydroxychloroquine	6	20
Immunosuppressant (CsA, MTX, MMF)	23	77
Rituximab	2	6
Organ involvement		
Interstitial lung disease	9	30
Clinically overt heart involvement	11	37
Dysphagia	15	50
Arthritis	9	30
Median follow-up period (min–max) (From 20% SCIg start to the last visit; months)	87 (12–148)	

CsA, Cyclosporin A; MTX, Methotrexate; MMF, Mycophenolate mofetil; SCIg, Subcutaneous Immunoglobulin.

The median duration of 20% SCIg treatment was 42 months (min–max: 7–112 months).

Five out of 30 patients received two distinct cycles of SCIg at 3–5 years between one cycle and another. Five out of 30 patients were still on SCIg therapy at the end of the study period, of whom two were in their second cycle.

#### 3.1.1 Effectiveness Parameters

Overall, serum CK levels were significantly reduced after 20% SCIg treatment (p<0.001). The muscle strength was improved, with the median MMT8 score significantly increased after the treatment (p<0.001). The four patients suffering from dropped head syndrome due to severe weakness of neck extensor muscles improved dramatically after treatment.

Complete and partial skeletal muscle remission was documented in 12 and 18 patients, respectively, with no differences among PM and DM.

Before the initiation of 20% SCIg treatment, all the enrolled DM patients showed multiple skin events as heliotrope rash, periungual erythema, and skin psoriasis. Of them, 10 reported an improvement of skin condition after the treatment, one reported a worsening, whereas two patients remained stable.

The parameters related to the pulmonary function were comparable between the pre- and post-treatment evaluations (pre-treatment mean DLCO: 44% [range: 29–75%]; post-treatment mean DLCO: 48% [range: 28–68%], n=11. Pre-treatment mean FVC: 74% [range: 60–98%]; post-treatment mean FVC: 79% [range: 62–110%], n=11).

High-resolution chest CT documented interstitial lung disease in nine patients, which improved in two of them after combined treatment with glucocorticoid, 20% SCIg and immunosuppressant (methotrexate and rituximab respectively).

Clinically overt cardiac involvement was documented in 11 (36%) patients, ranging from arrhythmic disorders (n=1, 3%) and myopericarditis (n=3, 10%) to non-ischemic cardiomyopathy (n=7, 23%). Heart disease progressed in six patients (20%), with exitus in two of them, despite aggressive treatment. Finally, four patients (13%) had pulmonary arterial hypertension.

A significant improvement in dysphagia was reported after 20% SCIg treatment in nine out of 15 patients. Pre-treatment mean DOSS increased from 5.0 (range: 3.0–5.0) to post-treatment mean DOSS 6.0 (range: 5.0–7.0, p=0.002).

Nine patients (30%) presented with arthritis before the treatment. We documented stable, improved or worsened disease in 5 (16%), 2 (6%) and 2 (6%) patients, respectively. Three DM female patients (10%) had associated neoplasia (thyroid, breast, and vulvar cancers).

**Table 2** shows mean MITAX values at the start of SCIg treatment, which improved significantly, as documented at the last evaluation visit (p=0.022). Even if no significant changes were reported for mean MDI scores, HAQ-DI scores significantly improved after treatment (p=0.002).

**Table 2:** Selected parameters before and after 20% SCIg treatment in patients with PM and DM.

Parameters	n	Pre-treatment; median (min-max)	Post-treatment; median (min-max)	p-value
CK levels	30	884 (33–1,525)	104 (24–800)	<0.001
MMT8	29	67 (46–78)	78 (48–80)	<0.001
PDN, mg*	30	25 (5–100)	4 (0–25)	<0.001**
***Disease activity***				
MITAX	26	0.11 (0–0.52)	0.09 (0–0.32)	0.022
***Assessment of damage***				
MDI	28	0.09 (0–1.04)	0.14 (0–0.90)	0.100
***Assessment of disability***				
HAQ-DI	15	0.31 (0–3.0)	0.66 (0–3.0)	0.002

* Mean previous versus current daily prednisone-equivalent dose.

** Wilcoxon non-parametric test.

As for glucocorticoid therapy, the median prednisone-equivalent dose after the treatment was 3.8 mg/day (range 0–25 mg/day), which is significantly lower than the mean prednisone-equivalent dose before the treatment (25 mg/day; range: 5–100 mg/day; **Table 2**, p<0.001). Seven patients (23%) withdraw prednisone after 20% SCIg therapy.

Fourteen patients (60%) withdrew from the immuno-suppressant at the end of the 20% SCIg therapy; this reduction was significant (p<0.001).

The selected parameters were compared between patients previously treated with IVIg (n=19) versus patients who started Ig therapy with 20% SCIg therapy (n=11). For any indicator, we did not detect any difference between the two groups at the end of 20% SCIg therapy.

#### 3.1.2 Safety Data

Three death events unrelated to SCIg therapy were reported during the study period (10% of patients). These were caused by cardiovascular complications in two cases and COVID-19 in one case. None of the remaining patients reported systemic reactions to the 20% SCIg preparation, and none discontinued the treatment. Local reactions were evaluated on 27 patients and were erythema (n=16, 53%), swelling (n=9, 30%) and nodule (n=2, 6%) (**Table 3**). In most cases, the duration of local reactions was less than 30 minutes after injection (n=14, 47%). The duration was a few hours for 10 patients (33%) and 1 day for three patients (10%). All the local reactions resolved spontaneously.

**Table 3:** Comparison between local adverse events in PM/DM and CVID patients.

	PM/DM, n (%)	CVID, n (%)	p-value
			0.275
Erythema	16 (53)	9 (31)	
Erythema + swelling	0 (0)	6 (7)	
Swelling	9 (30)	10 (35)	
Erythema + swelling + subcutaneous nodules	0 (0)	1 (3)	
Subcutaneous nodules	2 (6)	1 (3)	
None	3 (10)	6 (21)	

#### Patient Satisfaction

**Table 4** shows the results of the satisfaction questionnaire related to the use of 20% SCIg. Overall, most DM/PM patients reported a well-tolerated use of the 20% SCIg treatment.

**Table 4:** Satisfaction data in the 27 PM/DM surviving patients.

Satisfaction data	n=27; n (%)
Opinion about the experience with the 20% SCIg treatment:	
• Good	14 (47)
• Very good	13 (43)
Opinion about the training period:	
• Good	14 (47)
• Very good	13 (43)
Difficulty in preparing the infusion:	
• No difficulty	23 (77)
• NA	4 (15)
Support from the healthcare staff:	
• Yes	24 (80)
• No	2 (7)
• NA	1 (4)
During the infusion, patients reported:	
• To stay still	14 (47)
• To walk	7 (23)
• To do small jobs	6 (20)

NA, not available. All patients receive the infusion in the abdomen.

### 3.2 CVID Patients

Data of 29 CVID patients were evaluated. All the baseline characteristics of CVID patients are reported in **Supplementary Table 1**. Recurrent respiratory infections, including upper respiratory infection (URI), lower respiratory infection (LRI) and sinusitis, were present in almost all patients (28/29, 96%). In 15 (51%) patients, URI or LRI were the only features of the CVID, while in 13 (44%) patients, at least two concomitant respiratory infections were present (**Supplementary Table 1**). Fourteen patients (48%) had chronic lung disease with bronchiectasis. Ten patients (34%) had infections only (“not complicated” phenotype), whereas the remaining patients presented with a “complicated phenotype”. Autoimmune diseases were present in 11 patients (38%), mostly immune thrombocytopenia (ITP; n= 6, 20%) and polyautoimmunity (n=5, 17%). Finally, 12 patients (41%) had polyclonal lymphoproliferation, 7 (24%) enteropathy, and 6 (21%) a neoplasm.

Nine (31%) CVID patients were treated with 20% SCIg as the first Ig treatment, whereas 20 patients (69%) switched to 20% SCIg after IVIg treatment.

The median duration of 20% SCIg treatment was 56 months (min–max: 12–150 months) at a weekly dose of 8 g in 72% of patients (n=21) and 6 g in the remaining patients (n=8, 28%). The standard treatment was followed by 16 patients (55%), whereas 8 (28%) patients followed a seasonal modified regimen, which extends the dosing interval in summer months. Due to severe enteropathy, one patient (3%) underwent a combination treatment (20% SCIg + IVIg). Twenty-two (76%) patients were still on SCIg therapy at the study cut-off date.

#### 3.2.1 Effectiveness Parameters

After 20% SCIg therapy, a significant improvement was observed for all the investigated parameters (**Table 5**).

**Table 5:** Selected parameters before and after 20% SCIg treatment in patients with CVID (n=29).

	Pre-treatment; median (min–max)	Post-treatment; median (min–max)	p-value*
IgG	347 (24–618)	875 (326–1250)	**<0.001**
No. of infections	5.5 (2–9)	0.5 (0–3.5)	**<0.001**
Patients with serious infections, n (%)	21 (72)	3 (10)	<0.001**
No. of serious infections	1 (0–6)	0 (0–2)	**<0.001**
No. of antibiotics administration per year	4.5 (1–8)	0.5 (0–2.5)	**<0.001**
Hospitalizations (per year)	1 (1–4)	1 (0-1)	**0.001**
Hospitalized patients, n (%)	13 (43)	6 (21)	**0.050**
Days in hospital	7 (2–30)	4 (0–30)	**0.002**
Absence from work (days)	7 (2–30)	4 (3–5)	**0.010**

*Wilcoxon non-parametric test; **McNemar non-parametric test.

Statistically significant p-value are reported in bold.

Considering the infection status, after 20% SCIg therapy, three out of 29 patients (10%) no longer have infections. In eleven patients (38%), the severity of infections decreased, as seven patients (24%) went from a URI + LRI diagnosis to a URI-only diagnosis, and four patients (14%) went from LRI to URI diagnosis.

The diagnostic delay, categorized as ≤10 years versus >10 years, did not affect any of the indicators mentioned in **Table 5**.

The selected parameters were compared between patients previously treated with IVIg (n=20) versus patients treated with 20% SCIg as the first Ig treatment (n=9). For any indicator, no significant differences were found between the two groups at the end of the 20% SCIg therapy.

An additional analysis was performed comparing CVID patients with a “not complicated” phenotype (n=10, 34%) to those with a “complicated phenotype” (n=19, 66%), In this subgroup of patients, a significant improvement was observed for all the investigated parameters except for the number of hospitalized patients, which reduction was not statistically significant after the therapy (**Supplementary Table 2**). The complementary analysis performed on patients with “not complicated” phenotype showed that the hospitalized patients, the days in hospital and absences from work were not significantly reduced after the treatment (**Supplementary Table 3**).

#### 3.2.2 Safety Data

**Table 3** summarizes the adverse events (AEs) observed in CVID patients after the treatment with 20% SCIg. All AEs were of a mild entity and self-limiting. Of note, two patients received 20% SCIg therapy (tolerated) after a not tolerated IVIg therapy. Four patients stopped SCIg therapy and switched to IVIg therapy (one for aesthetic reasons, three for poor compliance). Three death events unrelated to the SCIg therapy were reported during the study period (10% of patients). In one case, these were caused by a cerebral hemorrhage and were related to a gastric and a pancreatic cancer in the other two cases.

#### 3.2.3 Patient Satisfaction

Results of the satisfaction questionnaire related to the treatment with 20% SCIg are summarized in **Table 6**. Overall, a well-tolerated use of 20% SCIg treatment was reported by the majority of CVID patients.

**Table 6:** Satisfaction data in CVID patients.

Satisfaction data	n=27, n (%)
Opinion about the experience with the 20% SCIg treatment:	
• Very good	18 (62)
• Good	7 (24)
• Sufficient	2 (7)
Opinion about the training period:	
• Very good	11 (38)
• Good	16 (55)
Difficulty in preparing the infusion:	
• No difficulty	27 (93)
Support from the healthcare staff:	
• Yes	27 (93)
During the infusion, patients reported:	
• To stay still	10 (34)
• To walk	2 (7)
• To do small jobs	15 (52)

All patients receive the infusion in the abdomen.

## 4 Discussion

The experience of our center shows the beneficial effects in terms of immunomodulatory and anti-inflammatory activities and the safety of long-term 20% SCIg administration in PM and DM patients.

Overall, after a median follow-up of 87 months, the CK levels were significantly reduced in these patients after the treatment, compared with before the initiation of therapy (p<0.001). The biological reduction of serum CK levels is mirrored by the clinical improvement in muscle strength and the resolution of dysphagia, as documented by the significant increase in MMT8 and in the DOSS scale (p<0.001 and p=0.002, respectively) and in line with previous data that related SCIg therapy with an improvement in dysphagia (38). Of note, our study reports the first evaluation of MITAX, MDI and HAQ-DI parameters after 20% SCIg treatment.

Although the mechanism of action is still to be clarified, different hypotheses have been formulated to explain the immunomodulatory activity of Ig in autoimmune diseases, such as the anti-idiotype regulation, modifications in cytokine production, inhibition of complement activation, neutralization of autoantibodies, killing of target cells by antibody-dependent cytotoxicity and the blockade of cell–cell interaction. Part of these mechanisms is mediated by the Fc-dependent pathways, which comprise the accelerated clearance of pathogenic antibodies by the saturation of the neonatal Fc receptor, the expansion of regulatory T cells, and the blockade of immune complexes (39–41). In particular, the therapeutic benefits of SCIg therapy in myositis patients could be linked to the administration route with Ig used at low dosages (<1 g/kg/monthly), that guarantees serum IgG steady-state levels which in turn probably influence chronic mechanisms of damage, such as regulation of T regulatory activity and dendritic cells functions (30, 42). As reported in previous studies, the role of T-regulatory cells in autoimmune diseases can be linked to their action in suppressing the activity of self-reactive T cells, contributing to the prevention of autoimmune phenomena (30). This hypothesis could be supported by the long-term evaluation of disability (as reflected by the improvement in HAQ-DI scores). In contrast, the index reflecting the activity of the disease is less impacted by 20%SCIg treatment (less reduction in MITAX scores). Therefore, in the active phase of the disease, it is better to use a more aggressive induction therapy based on glucocorticoid, immunosuppressant and IVIg, whereas the remission could be successfully maintained by the chronic use of 20% SCIg (42, 43).

The use of 20% SCIg in our study was also associated with an important steroid and immunosuppressant sparing effect, further explaining the improvement in HAQ-DI scores. None of the patients reported systemic reactions to the therapy. The duration of the reported local reactions was less than 30 minutes, and all resolved spontaneously. For instance, the frequency of the adverse events reported by PM/DM patients was not different from what reported in CVID patients, where the use SCIg therapy is more consolidated. The results of the satisfaction questionnaire administered to PM/DM patients suggest a good tolerability profile of 20% SCIg therapy.

Within this study, the long-term effectiveness of 20% SCIg therapy was also evaluated in a cohort of CVID patients. A significant improvement was observed for all the considered parameters at the end of the treatment. In these patients, the treatment was effective even in the case of modified therapeutic regimens (e.g., the seasonal regimen), underlining the versatility of 20% SCIg (44, 45). Of note, the analysis of the effectiveness parameters in a subgroup of CVID patients with a complicated phenotype suggested the relevant impact of the 20% SCIg therapy in these patients, with a consequent improvement in their quality of life.

Our data show that serum IgG levels have more than doubled at the end of 20% SCIg treatment in CVID patients. In line with previous data, the achievement of a sustained IgG serum level after 20% SCIg therapy shows to protect patients from recurrent infections, as supported by the significant reduction of days of hospitalization and work absence (9, 46). This also suggests a good adherence to effective dosing and administration in these patients.

The effect of the Ig therapy at replacement dosage on non-infectious concomitant co-morbidities (autoimmunity, polyclonal lymphoproliferation, and enteropathy) are not fully elucidated. All our CVID patients presented with autoimmune disease before the initiation of 20% SCIg therapy, and therefore it was not possible to evaluate the immunomodulatory effect of SCIg treatment in this setting. No patient showed relapses of autoimmune disease during 20% SCIg therapy, except for a patient with recurrence of myelitis and one with the onset of ex novo hepatic granulomatosis (considered as a form of polyclonal lymphoproliferation).

For instance, literature suggests that in patients with specific co-morbidities, such as protein-losing enteropathy, the treatment with SCIg may result in more stable IgG levels compared to IVIg therapy (47).

In our experience, cancer represents the first cause of death in CVID patients. A role for IVIg therapy in the treatment of cancer and its metastases has been suggested in previous studies (48, 49), while no evidence is available on SCIg. It has been suggested that the administration of IVIg supplemented with SCIg can support the cancer treatment, but more evidence is needed to confirm this preliminary observation (50).

Long-term tolerability is a fundamental issue to those with chronic diseases, such as CVID, as treatment is expected to extend throughout a patient’s lifetime. None of our patients reported systemic reactions, and none of them discontinued the treatment. Only local self-limiting AEs were reported, mainly swelling and erythema. It is noteworthy that various reports described the safe use of SCIg in patients with previous serious systemic AEs to IVIg, along with a better-tolerated profile of SCIg (51, 52). In line with this observation, two patients in our cohort of CVID patients tolerated the 20% SCIg therapy after not tolerating IVIg.

Previous studies evaluated the treatment satisfaction with 20% SCIg therapy, showing a significant improvement in the domain ‘Convenience’ in patients switching from IVIg and sustained treatment satisfaction in patients switching from another SCIg regimen, suggesting favorable effects on patients’ quality of life (9, 53). Within this study, the results of the satisfaction questionnaire administered to CVID patients support this evidence.

Even if this study presents some limitations, as the observational nature in a single-center context and the small population, it suggests the feasibility, effectiveness, and tolerability of 20% SCIg therapy in patients with DM/PM offering a valid therapeutic alternative to IVIg with important advantages for the quality of life of patients, especially those with difficult venous access, with unsatisfactory clinical response, and in patients preferring home care administration. Moreover, this study suggests that 20% SCIg therapy represents an important therapeutic alternative to the use of immunosuppressants: therapy with SCIg is, in fact, linked to a lower risk of infections, leading to a global improvement in the quality of life of patients.

## Data Availability Statement

The raw data supporting the conclusions of this article will be made available by the authors, without undue reservation.

## Ethics Statement

The studies involving human participants were reviewed and approved by Comitato Etico Regione Marche (CERM). The patients/participants provided their written informed consent to participate in this study.

## Author Contributions

Study conception and design: MGD. Data collection: JUV and CM. Statistical analysis: IT. Interpretation of data: MGD, JUV, CM, SS, and MBB. Manuscript drafting and editing: MGD. Manuscript approval to submit: MGD, JUV, CM, IT, SS, MBB, and GM. All authors contributed to the article and approved the submitted version.

## Funding

This study was supported by internal funds. This study received funding from CSL Behring Spa (Milan, Italy) only for the Editorial assistance. The funder was not involved in the study design, collection, analysis, interpretation of data, the writing of this article or the decision to submit it for publication.

## Conflict of Interest

The authors declare that the research was conducted in the absence of any commercial or financial relationships that could be construed as a potential conflict of interest.

## Publisher’s Note

All claims expressed in this article are solely those of the authors and do not necessarily represent those of their affiliated organizations, or those of the publisher, the editors and the reviewers. Any product that may be evaluated in this article, or claim that may be made by its manufacturer, is not guaranteed or endorsed by the publisher.
