# Supplementary material for: Replacement and Immunomodulatory Activities of 20% Subcutaneous Immunoglobulin Treatment: A Single-Center Retrospective Study in Autoimmune Myositis and CVID Patients
Source: Front Immunol. 2022 Jan 17;12:805705. doi: 10.3389/fimmu.2021.805705 (PMC8801806; doi:10.3389/fimmu.2021.805705)
Supplement: Supplementary file 1 [file DataSheet_1.docx]

**Replacement and immunomodulatory activities of 20% subcutaneous immunoglobulin treatment: a single-center retrospective study in myositis and CVID patients**

| **Supplementary Table 1.** Baseline characteristics of CVID patients (n=29) and clinical phenotypes according to Chapel et al. [37] | |
| --- | --- |
| **Baseline characteristics^§^** | **n (%)** |
|  |  |
| Gender: Female | 18 (62) |
| Age at disease onset (years), median (min–max) | 30 (8–67) |
| Age at diagnosis (years), median (min–max) | 46 (16–71) |
| Immunoglobulin at diagnosis, median (min–max) |  |
| IgA | 24 (3–166) |
| IgG | 347 (24–618) |
| IgM | 34 (4–203) |
| Recurrent respiratory infections | 28 (96) |
| URI | 6 (22) |
| LRI | 9 (32) |
| URI + sinusitis | 2 (7) |
| URI + LRI | 7 (25) |
| Sinusitis + LRI | 2 (7) |
| URI + sinusitis + LRI | 2 (7) |
| Bronchiectasis* | 14 (48) |
| Infections only (not complicated phenotype) | 10 (34) |
| Autoimmunity | 11 (38) |
| Organ-specific disease (myelitis, IDDM, vitiligo) | 3 (10) |
| Immune thrombocytopenic purpura | 6 (21) |
| Systemic disease (systemic sclerosis, Sjogren’s syndrome) | 2 (33) |
| Policlonal lymphoproliferation | 12 (41) |
| Granulomatosis | 6 (50) |
| Granulomatosis category | 6 (50) |
| 1 site | 2 (16) |
| 1+ sites | 4 (34) |
| Enteropathy | 7 (24) |
| Neoplasms | 6 (21) |
| LNH | 2 (33) |
| Other neoplasms (stomach, pancreas, breast, skin) | 4 (66) |
| Splenectomy | 3 (10) |
| Allergies | 9 (31) |
| Drugs | 7 (78) |
| Antigens | 1 (11) |
| Contrast media | 1 (11) |
| Family history | 9 (31) |
| Immunodeficiency | 3 (33) |
| Immunodeficiency + autoimmunity | 5 (56) |
| Cancer | 1 (11) |
| Smokers | 8 (28) |
| Mean follow-up period (from SCIg start to last visit; months) | 56 (10–150) |
| *18 patients showed concomitant conditions: n=2 URI + bronchiectasis, n=3 LRI + bronchiectasis, n=1 URI + sinusitis, n=1 URI + sinusitis + bronchiectasis, n=3 URI + LRI, n=4 URI + LRI + bronchiectasis, n=1 URI + sinusitis + LRI, n=1 URI + sinusitis + LRI + bronchiectasis, n=2 SINU + LRI + bronchiectasis.  ^§^17 patients had respiratory pathologies concomitant to other pathologies as follows: n=6 with cancer, n=5 with gastrointestinal disease, n=4 with granulomatosis, n=1 gastrointestinal disease + granulomatosis + cancer, n=1 gastrointestinal disease + granulomatosis.  URI: upper respiratory infection; LRI: lower respiratory infection; IDDM: insulin-dependent diabetes mellitus; CVID: common variable immunodeficiency; SCIg: subcutaneous immunoglobulin. | |

| **Supplementary Table 2.** Selected parameters before and after 20% SCIg treatment in patients with CVID and a complicated phenotype (n=19). | | | |
| --- | --- | --- | --- |
|  | Pre-treatment; median (min–max) | Post-treatment; median (min–max) | p-value* |
| IgG | 368 (24-548) | 800 (326-1040) | <0.001 |
| No. of infections | 4.5 (2-9) | 1.0 (0-3.5) | <0.001 |
| Patients with serious infections, n (%) | 14 (78) | 3 (100) | 0.001 |
| No. of serious infections | 2 (0-6) | 0 (0-2) | 0.001 |
| No. of antibiotics administration per year | 4.5 (1-7.5) | 0.5 (0-1.5) | <0.001 |
| Hospitalizations (per year) | 2 (1-4) | 1 (0-1) | 0.001 |
| Hospitalized patients, n (%) | 15 (79) | 10 (53) | 0.267 |
| Days in hospital | 11.3 (4-30) | 4 (0-30) | 0.033 |
| Absence from work (days) | 10.3 (0-30) | 4 (3-5) | 0.008 |
| *Wilcoxon non-parametric test; ** McNemar non-parametric test.  Statistically significant p-value are reported in bold. | | | |

| **Supplementary Table 3.** Selected parameters before and after 20% SCIg treatment in patients with CVID and a not complicated phenotype (n=10). | | | |
| --- | --- | --- | --- |
|  | Pre-treatment; median (min–max) | Post-treatment; median (min–max) | p-value* |
| IgG | 282 (37-618) | 881 (607-1250) | 0.005 |
| No. of infections | 5.5 (2-9) | 0.5 (0-2.5) | 0.005 |
| Patients with serious infections, n (%) | 7 (78) | 0 | not estimable** |
| No. of serious infections | 1 (0-3) | 0 (0) | 0.011 |
| No. of antibiotics administration per year | 5.5 (1-8) | 0.5 (0-2.5) | 0.005 |
| Hospitalizations (per year) | 1 (1-4) | 0.5 (0-1) | 0.014 |
| Hospitalized patients, n (%) | 6 (60) | 5 (50) | 1.000** |
| Days in hospital | 3.8 (2-15) | 3.8 (0-5) | 0.593 |
| Absence from work (days) | 3.7 (2-7) | 4.4 (3.5-5) | 0.655 |
| *Wilcoxon non-parametric test; ** McNemar non-parametric test.  Statistically significant p-value are reported in bold. | | | |
